# Supplementary material for: Placenta Previa Complicated with Endometriosis: Contemporary Clinical Management, Molecular Mechanisms, and Future Research Opportunities
Source: Biomedicines. 2021 Oct 26;9(11):1536. doi: 10.3390/biomedicines9111536 (PMC8614896; doi:10.3390/biomedicines9111536)
Supplement: Supplementary file 1 [file biomedicines-09-01536-s001.zip › Supplemental Files S1-S2.pdf]

Supplemental File S1. The search strategy for the study about the diagnosis of endometriosis during pregnancy.

PubMed
#1 Endometriosis [MeSH]
#2 endometriosis [TIAB] OR endometrioma [TIAB] OR chocolate cyst [TIAB]
#3 adhesion [TIAB] OR “ectopic endometri*” [TIAB]
#4 peritoneal [TIAB] OR abdominal [TIAB] OR pelvic [TIAB] OR Douglas [TIAB] OR “uterine wall” [TIAB] OR “anterior wall” [TIAB] OR “posterior wall” [TIAB] OR abdominal [TIAB] OR ovary [TIAB] OR extrauterine [TIAB] OR extraovarian [TIAB] OR cul-de-sac [TIAB] OR adnexal [TIAB]
#5 #3 AND #4
#6 #1 OR #2 OR #5
#7 Epidemiology [MeSH] OR Interrupted Time Series Analysis [MeSH]
#8 National [TIAB] OR nationwide [TIAB] OR “population-based”
#9 #7 OR #8
#10 #5 AND #9

Cochrane Library
#1 MeSH descriptor: [Endometriosis] explode all trees
#2 endometriosis:ab,ti,kw OR “endometrioma:ab,ti,kw OR “chocolate cyst”:ab,ti,kw
#3 #1 OR #2
#4 adhesion:ab,ti,kw OR ectopic endometrium:ab,ti,kw
#5 peritoneal:ab,ti,kw OR abdominal:ab,ti,kw OR pelvic:ab,ti,kw OR Douglas:ab,ti,kw OR “uterine wall” :ab,ti,kw OR “anterior wall”:ab,ti,kw OR “posterior wall”:ab,ti,kw OR abdominal:ab,ti,kw OR ovary ab,ti,kw OR extrauterine:ab,ti,kw OR extraovarian:ab,ti,kw OR cul-de-sac:ab,ti,kw OR adnexal :ab,ti,kw
#6 #4 AND #5
#7 #3 OR #6
#8 MeSH descriptor: [Epidemiology] explode all trees
#9 MeSH descriptor: [Interrupted Time Series Analysis] explode all trees
#10 National:ab,ti,kw OR nationwide:ab,ti,kw OR population-based:ab,ti,kw
#11 #8 OR #9 OR #10
#12 #7 AND #11

Scopus
#1 TITLE-ABS-KEY ("endometriosis" OR "endometrioma" OR “chocolate cyst”)
#2 TITLE-ABS-KEY ("adhesion" OR “ectopic endometrium”)

#3 TITLE-ABS-KEY (peritoneal OR abdominal OR pelvic OR Douglas OR “uterine wall” OR “anterior wall” OR “posterior wall” OR abdominal OR ovary OR extrauterine OR extraovarian OR cul-de-sac OR adnexal)
#4 #2 AND #3
#5 #1 OR #4
#6 TITLE-ABS-KEY ("epidemiology" OR "National" OR “nationwide” OR “population-based”)

Supplemental File S2. The search strategy for the study that examined the effect of placenta previa on the rate of placenta previa.

PubMed

#1 Endometriosis [MeSH]

#2 endometriosis [TIAB] OR endometrioma [TIAB] OR chocolate cyst [TIAB]

#3 adhesion [TIAB] OR “ectopic endometri*” [TIAB]

#4 peritoneal [TIAB] OR abdominal [TIAB] OR pelvic [TIAB] OR Douglas [TIAB] OR “uterine wall” [TIAB] OR “anterior wall” [TIAB] OR “posterior wall” [TIAB] OR abdominal [TIAB] OR ovary [TIAB] OR extrauterine [TIAB] OR extraovarian [TIAB] OR cul-de-sac [TIAB] OR adnexal [TIAB]

#5 #3 AND #4

#6 #1 OR #2 OR #5

#7 Placenta previa [MeSH]

#8 “placenta previa" [TIAB] OR "low lying placenta" [TIAB]

#9 "abnormal placenta*" [TIAB]

#10 Placenta accreta [MeSH]

#11 “Morbidly adherent placenta" [TIAB] OR "Morbid adherent placenta" [TIAB]

#12 “Placenta accreta spectrum " [TIAB]

#13 “Placenta accreta”[TIAB] OR "Placenta increta" [TIAB] OR "placenta percreta" [TIAB]

#14 "adherence of the placenta" [TIAB] OR "adherent placenta" [TIAB]

#15 #7 OR #8 OR #9 OR #10 OR #11 OR #12 OR #13 OR #14

#16 #6 AND #15

Cochrane Library

#1 MeSH descriptor: [Endometriosis] explode all trees

#2 endometriosis:ab,ti,kw OR “endometrioma:ab,ti,kw OR “chocolate cyst”:ab,ti,kw

#3 #1 OR #2

#4 adhesion:ab,ti,kw OR ectopic endometrium:ab,ti,kw

#5 peritoneal:ab,ti,kw OR abdominal:ab,ti,kw OR pelvic:ab,ti,kw OR Douglas:ab,ti,kw OR “uterine wall”:ab,ti,kw OR “anterior wall”:ab,ti,kw OR “posterior wall”:ab,ti,kw OR abdominal:ab,ti,kw OR ovary:ab,ti,kw OR extrauterine:ab,ti,kw OR extraovarian:ab,ti,kw OR cul-de-sac:ab,ti,kw OR adnexal:ab,ti,kw

#6 #4 AND #5

#7 #3 OR #6

#8 MeSH descriptor: [Placenta previa] explode all trees

#9 “Placenta previa":ab,ti,kw OR "low lying placenta":ab,ti,kw

#10 "abnormal placenta":ab,ti,kw

#11 MeSH descriptor: [Placenta accreta] explode all trees
#12 “Morbidly adherent placenta":ab,ti,kw OR "Morbid adherent placenta":ab,ti,kw
#13 “Placenta accreta spectrum":ab,ti,kw
#14 "Placenta accreta":ab,ti,kw OR "Placenta increta":ab,ti,kw OR "Placenta percreta":ab,ti,kw
#15 "adherence of placenta":ab,ti,kw OR "adherence of the placenta":ab,ti,kw OR "adherent placenta":ab,ti,kw
#16 #8 OR #9 OR #10 OR #11 OR #12 OR #13 OR #14 OR #15
#17 #7 AND #16

Scopus
#1 TITLE-ABS-KEY ("endometriosis" OR "endometrioma" OR “chocolate cyst”)
#2 TITLE-ABS-KEY ("adhesion" OR “ectopic endometrium”)
#3 TITLE-ABS-KEY (peritoneal OR abdominal OR pelvic OR Douglas OR “uterine wall” OR “anterior wall” OR “posterior wall” OR abdominal OR ovary OR extrauterine OR extraovarian OR cul-de-sac OR adnexal)
#4 #2 AND #3
#5 #1 OR #4
#6 TITLE-ABS-KEY (“placenta previa” OR “low lying placenta”)
#7 TITLE-ABS-KEY (“abnormal placentation”)
#8 TITLE-ABS-KEY ("Morbidly adherent placenta" OR "Morbid adherent placenta")
#9 TITLE-ABS-KEY ("Placenta Accreta Spectrum")
#10 TITLE-ABS-KEY (“Placenta accreta” OR "Placenta increta" OR "Placenta percreta")
#11 TITLE-ABS-KEY ("adherence of placenta" OR "adherence of the placenta" OR "adherent placenta")
#12 #6 OR #7 OR #8 OR #9 OR #10 OR #11
#13 #5 AND #12
